# The binding mechanism of a novel ferrous ion chelating peptide from chicken blood hemoglobin and the bioavailability of the chelate

**DOI:** 10.1016/j.fochx.2025.103349

**Published:** 2025-11-29

**Authors:** Hanyu Guo, Ying Zhou, Cancan Luo, Zhiyu Li, Jiulan Peng, Weimin Xu, Daoying Wang, Jing Yang

**Affiliations:** a Institute of Agro-product Processing, Jiangsu Academy of Agricultural Sciences, Nanjing, Jiangsu 210014, PR China; b School of Life Sciences, Anhui Normal University, Wuhu, Anhui 241001, PR China; c School of Life Sciences and Food Engineering, Huaiyin Institute of Technology, Huaian, Jiangsu 223003, PR China.

**Keywords:** Chicken blood hemoglobin, Ferrous ion chelating peptide (TAEDKKLIQ), Binding mechanism, Ferrous-peptide chelate (TAEDKKLIQ-Fe), Bioavailability

## Abstract

In this study, chicken blood hemoglobin hydrolysates were subjected to separation and purification. A novel peptide (TAEDKKLIQ) with high ferrous ion chelating activity was identified from chicken blood hemoglobin hydrolysate. The binding mechanism between TAEDKKLIQ and ferrous ions was elucidated using a combination of structural characterization, molecular docking, and molecular dynamics simulation. The results indicated that TAEDKKLIQ formed a monodentate coordination bond with ferrous ions via the carboxyl group on the Asp side chain, exhibiting a single binding site. Furthermore, the stability and cellular activity experiments demonstrated that TAEDKKLIQ-Fe not only exhibited good chemical stability but also surpassed lactoferrin, the conventional iron supplement, in cellular activity. This study provided new scientific evidence for the application of peptides derived from chicken blood in food processing and nutritional fortification systems, and provided theoretical support for developing highly efficient and safe peptide-based iron supplements.

## Highlights

• A novel peptide was isolated from chicken hemoglobin and determined to be TAEDKKLIQ.
• The chelation ability of TAEDKKLIQ was high, with a chelation rate over 90 %.
• The carboxyl group of the Asp side chain provided the monodentate coordination.
• The chelation was facilitated by electrostatic interactions.
• TAEDKKLIQ-Fe exhibited excellent bioavailability.

## 1 Introduction

Iron is an essential trace element for living organisms, and it fulfills a significant function in physiological processes like oxygen storage and transport, immune regulation, and cell proliferation (Ding et al., 2025). Iron deficiency is one of the nutrient deficiency diseases that not only causes symptoms such as hair loss but may also weaken immune function and increase the risk of various immune diseases (Sun et al., 2020). Therefore, the development of safe, efficient, and highly bioavailable iron supplements has remained a significant research focus in the field of food science. Peptide ferrous chelates, as a new kind of iron supplement, have attracted extensive attention due to their ability to remarkably boost the stability, absorbability, and bioavailability of iron (Lin et al., 2021).

Peptides are highly safe, low-cost, fast-absorption, and low-energy consumption (Sun et al., 2020). It also has many useful biological activities (Du et al., 2022; Wang et al., 2024), including ferrous ion chelation activity. In recent years, screening peptide segments with high chelating activity from natural protein hydrolysates and studying their chelating mechanisms have become a research focus. Sun et al. (2020) showed high ferrous ion chelating activity for Antarctic Krill derived nonapeptide, and the main site of iron binding was the carboxyl, hydroxyl, and amino groups. The main binding sites were found to be carboxylate, amino and imidazole groups in the study of the ferrous ion chelating ability of quinoa protein peptides by Ding et al. (2025). Lin et al. (2021) isolated and purified collagen peptides from tilapia skin, resulting in peptides (TSCICP) that exhibited high chelating activity toward ferrous ions. The iron chelating sites of these peptides were respectively associated with the carboxyl group of Asp/Glu and the guanidinium group of Arg/Lys. In addition, the stability of chelates and their dissociation characteristics in the gastrointestinal environment directly affect the absorption efficiency of ferrous ions. Bovine hemoglobin peptide ferrous chelate and oat peptide ferrous chelate were proved to have good digestive stability and high bioavailability by in vitro digestive experiments, cellular experiments, and animal experiments (Lin et al., 2022; Zhang & Liu, 2022). Since food-borne peptide iron chelates are both functional and nutritive, it is widely recognized that peptide iron chelates are ideal new iron supplements with great potential for development.

However, among the numerous natural proteins available for preparing iron-chelating peptides, chicken blood stands out as a resource-rich slaughter byproduct with unique advantages. Chicken blood is rich in edible protein, and is easily processed and purified (Yang et al., 2024), making it an excellent source for producing small-molecule bioactive peptides. However, most chicken blood is discarded as a byproduct due to its dark color and strong odor, resulting in a waste of protein resources (Wang et al., 2024). Compared to proteins from other sources, chicken blood as a byproduct, does not require extraction from primary products, making it more cost-effective. However, research on ferrous iron chelate peptides derived from chicken hemoglobin remains limited.

Therefore, this study aimed to investigate chicken hemoglobin to obtain ferrous ion-chelating peptides and explored the binding mechanisms. A new ferrous ion chelating peptide was isolated and identified from chicken blood hemoglobin hydrolysate. The sequence was Thr-Ala-Glu-Asp-Lys-Leu-Ile-Gln (TAEDKKLIQ). Subsequently, its structure before and after chelation was characterized using zeta potential, particle size, and scanning electron microscopy. The binding mechanism of AEDKKLIQ to ferrous ions was determined using instrumental analytical methods like Fourier transform infrared spectroscopy and Isothermal titration calorimetry assays, combined with molecular docking and molecular dynamics simulations. Subsequently, the TAEDKKLIQ-Fe was evaluated for stability and tested for cellular activity to verify the bioavailability. These findings may provide the chemical basis and theoretical support for the high-value utilization of chicken blood hemoglobin in functional foods and nutritional fortification.

## 2 Materials and methods

### 2.1 Materials

Fresh chicken blood was sourced from Jiangsu Lihua Dairy Co., Ltd. Peptides were synthesized by Shanghai Qiangyao Co., Ltd. The other remaining reagents are all analytical grade.

### 2.2 Separation and purification of chicken blood hemoglobin peptide

#### 2.2.1 Hydrolysis of chicken blood hemoglobin

Chicken blood hemoglobin extraction was carried out using the method reported by (Yang et al., 2024). ACD anticoagulant was added to chicken blood and centrifuged (1800 ×*g*, 10 min). Chicken blood hemoglobin was extracted from precipitated red blood cells using the swelling method, followed by alkaline protease hydrolysis at 50 °C for 8 h. The supernatant was gathered via centrifugation to obtain chicken blood hemoglobin hydrolysate.

#### 2.2.2 Ultrafiltration

The hydrolysate was classified into 3 fractions (>5 kDa, 3–5 kDa, and < 3 kDa) according to the size of molecular retention capacity by ultrafiltration membranes, and 3 fractions were collected for chelating rate determination.

#### 2.2.3 Sephadex G-25 gel filtration

The fraction with the highest chelating activity (4 mg/mL) was separated on a Sephadex G-25 column (100 cm × 2.6 cm). The eluent was ultrapure water at a flow rate of 1 mL/min, and the absorbance was measured at 214 nm. The separated peaks were collected to determine the ferrous chelating activity. The fraction showing the highest chelation rate was processed for subsequent purification.

#### 2.2.4 Reversed-phase high-performance liquid chromatography (RP-HPLC)

The component with the highest chelating activity (10 mg/mL) was purified by RP-HPLC. A C18 reversed-phase silica gel column (C18, 10 mm × 250 mm) was used, with eluent A (100 % acetonitrile) and eluent B (0.1 % formic acid aqueous solution). Under a gradient condition of 0–50 % B, elute at a flow rate of 1 mL/min for 45 min and monitor at a wavelength of 214 nm. The separated peaks were collected and their ferrous ion chelating activity was determined.

### 2.3 Determination of iron chelating rate

The detection of iron chelation activity was performed using the method described by Lin et al. (2021). The peptide solution was prepared using sodium acetate buffer. Subsequently, 1 mL of the peptide solution (1 mg/mL, pH 5) was mixed with 2 mL of FeCl₂•4H₂O (1.5 mM) and reacted at 37 °C for 30 min. Then, the alcohol precipitation method was used to determine the chelation rate of ferrous ions:

(1) $$\text{Iron chelating rate}\left(\%\right)=\frac{A-A_{1}}{A}\times 100\%$$

where: A is the total amount of ions added; A_1_ is the content of free ions in the supernatant.

### 2.4 Characterization of peptide sequences

The peptides were analyzed by LC-MS/MS (EASY-nLC 1200, Thermo Scientific, Waltham, MA, USA). Chromatographic separation was performed using an analytical column (C18, 75 μm × 15 cm, 3 μm, Thermo Scientific, Waltham, MA, USA), with elution using buffer B (80 % acetonitrile containing 0.1 % FA). The full scan mass spectrometry spectrum and MS/MS scan resolutions were 60,000 and 15,000, respectively. Peptide analysis was performed using Peaks Studio X Pro (Version 10.6) based on the UniProt database (Wang et al., 2025).

### 2.5 Verification of ferrous ion chelation activity

The peptides identified by mass spectrometry were synthesized by China Qiangyao Co., Ltd. (Shanghai, China) using Fmoc-protected amino acid solid-phase peptide synthesis, with the purity of these peptides evaluated at over 98 %. The ferrous ion chelating activity was verified using the method described in Section 2.3.

### 2.6 Preparation of TAEDKKLIQ-Fe chelate

The preparation of TAEDKKLIQ-Fe chelate was carried out according to the method described by Ding et al. (2025). The peptide with the highest chelation rate (TAEDKKLIQ) was chelated with ferrous ions under reaction conditions of 2.3. After the reaction was complete, separated the precipitate using alcohol precipitation. The resulting solid product was freeze-dried to obtain the TAEDKKLIQ-Fe chelate.

### 2.7 Structural characterization

#### 2.7.1 Zeta potential and particle size

An appropriate amount of TAEDKKLIQ and TAEDKKLIQ-Fe solutions with a concentration of 1 mg/mL were added to U-shaped cuvettes and equilibrated at a constant temperature of 25 °C for 5 s. The Zeta potential values and particle size distributions of the samples were determined using a Zetasizer Nano ZSE analyzer to evaluate their surface charge characteristics and solution stability.

#### 2.7.2 Thermogravimetry (TG)

5 mg of TAEDKKLIQ and TAEDKKLIQ-Fe lyophilized powders were weighed, respectively, and the thermal stability analysis was carried out by differential scanning calorimeter. The sample chamber temperature range is set from 50 to 800 °C, with a heating rate of 10 °C/min. The thermogravimetric change curves of the samples were recorded synchronously.

#### 2.7.3 Ultraviolet-visible (UV–vis) absorption spectroscopy

UV–Vis absorption spectroscopy was performed according to the method of Wei et al. (2024), with minor adjustments. TAEDKKLIQ solution (0.1 mg/mL) was reacted with FeCl₂•4H₂O (0.2 mM, 0.4 mM, 0.8 mM) in a reaction system at pH 8.0 and 50 °C for 60 min. The absorption spectra of the TAEDKKLIQ-Fe chelate and TAEDKKLIQ were measured in the wavelength range of 190–800 nm.

#### 2.7.4 Scanning electron microscopy (SEM)

Appropriate amounts of TAEDKKLIQ and TAEDKKLIQ-Fe powders were uniformly dispersed and fixed on both sides of the conductive tape on the sample stage, and then subjected to gold spraying and gold plating treatments. The samples were observed using a scanning electron microscope, and microscopic images of the sample surfaces were obtained to analyze the structural characteristics of the samples.

### 2.8 Binding mechanism

#### 2.8.1 Fluorescence spectroscopy (FS)

TAEDKKLIQ solution (0.2 mg/mL) was reacted with FeCl₂·4H₂O (1.0 mM, 1.5 mM, 2.0 mM, 2.5 mM) at pH 8 and 50 °C for 60 min. The fluorescence emission intensities of TAEDKKLIQ and TAEDKKLIQ-Fe were measured using fluorescence spectroscopy in the wavelength range of 290–400 nm, with an excitation wavelength of 295 nm (Wei et al., 2024).

#### 2.8.2 Fourier transform infrared (FTIR) spectroscopy

The lyophilized powdered samples of 2 mg TAEDKKLIQ and its iron chelate TAEDKKLIQ-Fe were accurately weighed, and after appropriate pre-treatment, the infrared spectral data were collected in the spectral range of 4000–400 cm-^1^, and the average value was taken by performing 32 replicate scans for each sample. The experimental data were processed and analyzed by OMNIC 8.2 software to resolve the functional group characteristics and structural changes of the samples.

#### 2.8.3 Isothermal titration calorimetry (ITC)

TAEDKKLIQ and FeCl₂•4H₂O were both soluble in Tris-HCl buffer (pH 7.4), and 1 mM Na₂S₂O₄ was added to the FeCl₂•4H₂O solution to maintain the ferrous ion in its divalent state. To ensure the accuracy of the experimental data, the solutions used in the experiments were filtered through 0.22 μm aqueous filtration membranes, and the degassing step was completed before the titration operation to effectively exclude the measurement bias that might be caused by air bubbles. In the experiment, the injection needle was loaded with FeCl₂•4H₂O solution, and the cuvette was filled with TAEDKKLIQ solution. The titration parameters were set as follows: 2 μL of FeCl₂•4H₂O solution per injection, 25 titrations in total, 200 s between injections, and the reaction temperature was 25 ± 0.2 °C. The experimental data were analyzed using nonlinear regression analysis with NanoAnalyze software, ultimately yielding a series of thermodynamic parameters such as the coordination number (n).

#### 2.8.4 Intermolecular force

Referring to the study by Sun et al. (2020), the intermolecular forces of TAEDKKLIQ-Fe were analyzed using different dissociation reagents. The absorbance values at 500 nm of the sample solution (2 mg/mL) were measured at the initial moment of the reaction (0 min) and after 30 min, respectively. At 30 min of the reaction, 1 mol/L NaCl, 30 mmol/L DTT (dithiothreitol), 0.5 % SDS, and 6 mol/L urea were added sequentially, followed immediately by absorbance detection using an enzyme meter.

#### 2.8.5 Molecular docking

The TAEDKKLIQ structure was constructed by Discovery Studio, and then the peptide structure was protonated at pH 7.4, followed by optimization of the peptide 3D structure to eliminate unfavorable spatial site-blocking conflicts using a multistage energy minimization approach, execution of 2000-step most rapid descent and 5000-step conjugate gradient algorithms for the three peptide structures mentioned above based on the implicit solvent model of GBMV. Energy minimization was performed, followed by molecular docking using CDOCKER based on a simulated annealing algorithm to explore the binding mode of Fe^2+^ to the above peptides (Ochoa & Fox, 2023).

#### 2.8.6 Molecular dynamics simulation

The peptides were typed using the CHARMM36 force field, and the ferrous ions were typed using the MATCH algorithm. Then the TIP3P water molecule model was placed around the orthogonal box (cell shape: Orthorhombic) to solvate the complex system using the orthogonal box (cell shape: Orthorhombic). The distance between the system and the boundary was set to 7 Å. The long-range LRI effect was calculated using PME (Particle Mesh Ewal), and a 0.145 M concentration of NaCl was used to neutralize the system's charge. All simulations were carried out under NPT system (constant pressure, constant temperature) periodic boundary conditions at 300 K. The distance cutoff used to count non-bonded interaction pairs was 14 Å, and the local interaction cutoff was 12 Å. In addition, the SHAKE was applied to constrain all covalent bonds involving hydrogen. After ensuring that equilibrium was reached after initial kinetic temperatures and energies, an unconstrained sampling phase of 200 ns was performed using NAMD (version 2.13-GPU), with temperature and pressure control using Lanzivan Dynamics and Lanzivan Piston, respectively. For the trajectory analysis of the complex system, monitoring the RMSD (Root Mean Square Deviation) of each frame compared to the first one and the interactions at the binding interface, which corresponds to the classical Euclidean distance between the two structures, is an effective tool to assess the stability of the whole trajectory of the system (Chen et al., 2025).

### 2.9 Stability analysis

#### 2.9.1 Temperature and pH stability test

The temperature stability of TAEDKKLIQ-Fe was determined using the method described by Qu et al. (2022). The aqueous solution of TAEDKKLIQ-Fe (1 mg/mL) was incubated for 2 h at a temperature gradient of 30–80 °C and a pH gradient of 2–9. TAEDKKLIQ-Fe was separated using the alcohol precipitation method to calculate the iron retention rate:

(2) $$\begin{array}{c} \text{Iron retention}\;\left(\%\right)=\frac{A-A_{0}}{A}\times 100\% \end{array}$$

where A represents the total iron content added to the reaction (mmol/mL), A_0_ represents the iron ion content in the supernatant after the reaction (mmol/mL).

#### 2.9.2 In vitro digestive simulation

In vitro simulated digestion of TAEDKKLIQ-Fe according to Ding et al. (2025). As an experimental control, ferrous sulfate and ferrous gluconate were also selected for parallel comparison in this study. Three iron supplements (TAEDKKLIQ-Fe, ferrous sulfate, and ferrous gluconate) were mixed to form a solution containing 2 mmol/mL of iron ions, which was used to simulate the digestive processes of the stomach, intestines, and gastrointestinal tract.

To simulate the digestive processes of the stomach and intestines separately, the pH values of the three iron supplements were adjusted to 2.0 and 7.6, respectively, and pepsin and trypsin (3 %, *w*/w) were added separately. The mixture was incubated in a water bath at 37 °C for 2 h. To simulate the digestive processes of the gastrointestinal tract, the three iron supplements were subjected to simulated gastric digestion followed by intestinal digestion. The retention rates of iron after gastric digestion, intestinal digestion, and simulated gastrointestinal digestion were determined according to the method described in Section 2.9.1.

#### 2.9.3 Effect of different dietary components

Following Kalgaonkar and Lönnerdal (2008), TAEDKKLIQ-Fe, ferrous gluconate, and ferrous sulfate solutions were preheated and equilibrated in a water bath at 37 °C. Then, the pH was adjusted to 2.0, and oxalic acid solution (1 %, *w*/*v*) was added. Gastrointestinal digestion simulation was then performed under the conditions described in 2.9.2. After the reaction was complete, the enzyme was inactivated, and the supernatant was collected after centrifugation. To assess the effects of phytate and dietary fiber on iron absorption, 1 % phytate and 8 % dietary fiber were added to the sample solution, and the digestion and measurement steps were repeated. By comparing changes in iron retention under different treatment conditions, the impacts of phytate and dietary fiber on the stability of TAEDKKLIQ-Fe chelate were analyzed. For calculations of iron retention rates, refer to Section 2.9.1.

### 2.10 Caco-2 cell viability assay

Caco-2 cell viability assay was conducted on TAEDKKLIQ-Fe according to the method of Wu et al. (2019). And lactoferrin was chosen as an experimental control. When the cell fusion rate was close to 80 %, the cells were dissociated and passaged using the trypsin-EDTA mixed digest. Cultivate the 23rd generation of cells in the logarithmic growth phase at 37 °C and 5 % CO₂ for 24 h. Then, a gradient concentration of TAEDKKLIQ-Fe and Ac-TAEDKKLIQ-Fe was added to the wells of the experimental group, and all the samples were incubated for 24 h. After incubation, 20 μL of MTT reagent was added to each well. After 2 h of reaction, 150 μL of DMSO was added and agitated for 10 min. The absorbance values at 490 nm were measured to reflect cell activity levels. Cell relative activity (%) was calculated:

(3) $$\begin{array}{c} \text{Relative cell viability}\;\left(\%\right)=\frac{A_{s}-A_{b}}{A_{c}-A_{b}}\times 100 \end{array}$$

where: As represents the absorbance value of the sample treatment, Ab represents the absorbance value without cells, and Ac represents the absorbance value without sample treatment.

### 2.11 Statistical analysis

All experimental data are presented as the mean ± standard deviation (Mean ± SD) from three independent experiments. Statistical analysis was performed via one-way ANOVA using SPSS 26.0 software (SPSS Inc., Chicago, USA), with Duncan's multiple range test applied to determine significance (*P <* *0.05*). Plotting was performed with OriginPro 2021 (OriginLab, USA).

## 3 Results and discussion

### 3.1 Separation and purification of chicken blood hemoglobin peptides

**Fig. 1:**
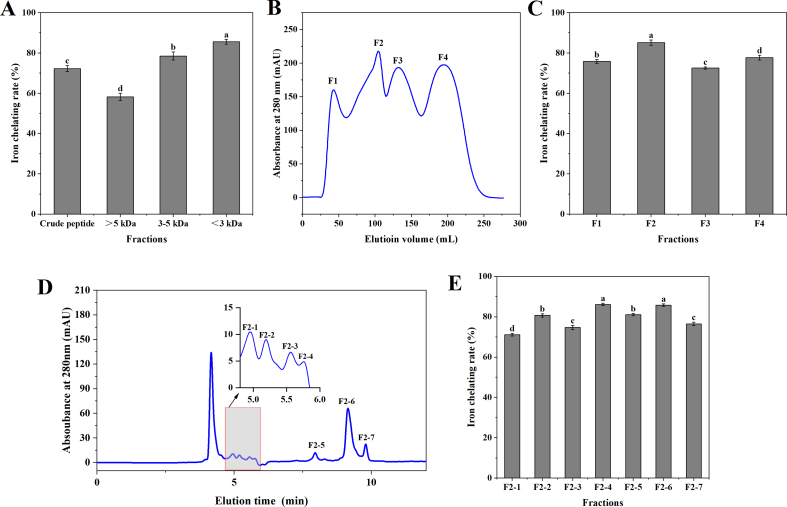
(A) Ferrous ion chelation rate of the four fractions after ultrafiltration separation. (B) Sephadex G-25 filtration chromatogram. (C) Ferrous ion chelation rate of four fractions obtained by Sephadex G-25 separation. (D) RP-HPLC. (E) Ferrous ion chelation rate of the seven fractions obtained by RP-HPLC separation. Data were shown as means ± SEM. Groups labeled with distinct letters are significantly different (*P <* *0.05*).

It is suggested that the molecular weight of peptides affects the ability to chelate iron (Sun et al., 2020). After ultrafiltration, chicken blood hemoglobin hydrolysate was divided into three fractions, and the iron chelation activity of each fraction was determined (Fig. 1A). The <3 kDa fraction had a chelating capacity of 85.56 %, which was significantly higher than the crude peptide, >5 kDa, and 3–5 kDa fractions (*P <* *0.05*). The study by Gao et al. (2024) showed that lower molecular-weight peptides exhibit a higher chelation rate. Therefore, the <3 kDa fraction was selected for subsequent purification stages.

The <3 kDa fraction was separated by gel chromatography, and four fractions, F1, F2, F3, and F4, were collected (Fig. 1B). Fig. 1C showed the iron chelating activity of each fraction. Among the obtained fractions, F2 had the highest iron chelating capacity of 85.12 %. Therefore, fraction F2 was used for the next step of purification.

After further separation of fraction F2 by RP-HPLC, seven fractions were collected as shown in Fig. 1D. By evaluating the iron chelating activities of these seven fractions, it was found that both F2–4 and F2–6 had higher iron chelating activities than the other fractions, 86.14 % and 85.81 %, respectively (Fig. 1E), but the results were not significant (*P >* *0.05*). Therefore, F2–4 and F2–6 were subsequently sequenced by LC-MS/MS.

### 3.2 Identification and verification of iron chelated peptides

The identified peptides were screened to remove peptides of microbial and enzymatic origin, and new peptide segments with a clear origin and a high average confidence score were selected (previously unreported). Six peptides were screened from the F2–4 and F2–6 fractions by LC-MS/MS. According to the UniProt database, all six peptides were identified as being from *Gallus* (chicken). The sequences, molecular weights, sources, and iron chelation rates of 6 peptides after synthesis are shown in Table 1. Molecular weights of six peptides ranging from 900 to 1200 Da. Lin et al. (2021) identified four iron-chelating peptides from tilapia skin collagen with molecular weights of 782.39–1441.68 Da, similar to the molecular weight distribution of 6 peptides in this study. High molecular weight peptides tend to aggregate in metal solutions, while low molecular weight peptides exhibit better water solubility, which facilitates iron chelation (Sun et al., 2020).

**Table 1:** Iron-chelated peptides identified from F2–4 and F2–6.

Number	Peptide sequence	MW (Da)	Chelation rate (%)	Protein name
1	KDYTPEVH	987.47	78.27 ± 0.31^d^	Hemoglobin subunit alpha-D(115th–122th)
2	TYPQTKTY	1000.49	59.75 ± 0.18^e^	Hemoglobin subunit alpha-D(35th–42th)
3	LTAEDKKLIQ	1157.67	81.18 ± 0.43^c^	Hemoglobin subunit alpha-D(2th–11th)
4	QAWEKAASH	1009.46	81.38 ± 0.27^c^	Hemoglobin subunit alpha-D(12th–20th)
5	TAEDKKLIQ	1044.58	90.94 ± 0.75^a^	Hemoglobin subunit alpha-D(3th–11th)
6	LSDLHAHKLR	1188.67	87.07 ± 0.12^b^	Hemoglobin subunit alpha-A(84th–93th)

Note: Data were shown as means ± SEM. Groups labeled with distinct letter are significantly different (P < 0.05).

Six peptides were synthesized, and the ferrous ion chelation rates were determined. Among them, the chelation rates of TAEDKKLIQ showed the highest chelation rate of 90.94 % ± 0.7 %. Therefore, the peptide with the best chelating activity (TAEDKKLIQ) was studied for the chelating mechanism with ferrous ions.

### 3.3 Structure characterization of TAEDKKLIQ and TAEDKKLIQ-Fe

#### 3.3.1 Zeta potential and particle size analysis

**Fig. 2:**
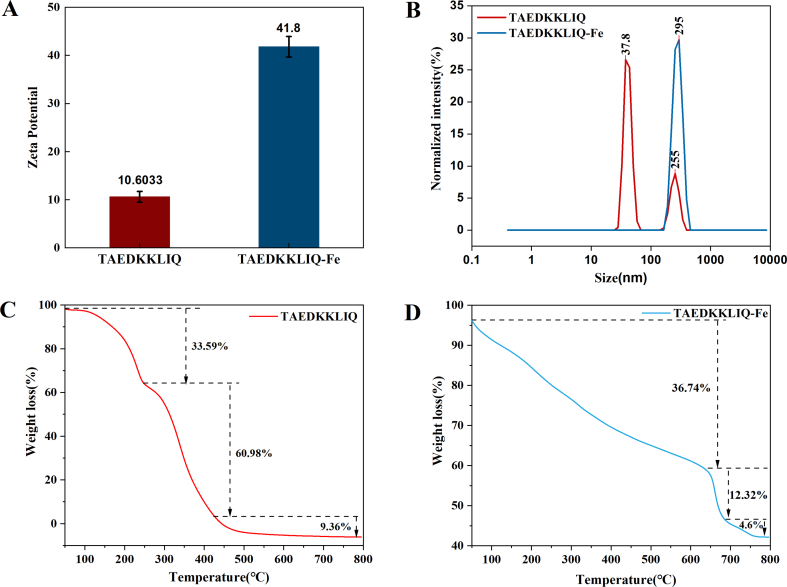
(A) Zeta potential of the TAEDKKLIQ and the TAEDKKLIQ-Fe chelate. (B) Particle size distribution. (C) TG-DSC of TAEDKKLIQ. (D) TG-DSC of TAEDKKLIQ-Fe. Data were shown as means ± SEM.

Zeta potential can be determined by the partial ionization of different amino acid residues in the peptide chain due to the surface charge of the sample (Lin et al., 2021). The zeta potential of TAEDKKLIQ and TAEDKKLIQ-Fe is shown in Fig. 2A. Compared to TAEDKKLIQ, the potential of TAEDKKLIQ-Fe was increased (*P < 0.05*), rising from 10.60 mV to 41.8 mV. Ferrous ions neutralized the negative charges on the peptide surface, causing an increase in charge, indicating that electrostatic interactions existed in the chelation reaction (Athira et al., 2021).

By analyzing the spatial distribution characteristics of the scattered light, the particle size distribution of the sample and its related parameters can be accurately determined (Lin et al., 2021). As shown in Fig. 2B, two peaks appeared for TAEDKKLIQ and one peak appeared for TAEDKKLIQ-Fe. The peaks of TAEDKKLIQ mainly appeared at 37.8 nm and 225 nm, and the peak of TAEDKKLIQ-Fe mainly appeared at 295 nm. This showed that chelation leads to an increase in average particle size. Research indicated that chelation alters the spatial structure of peptide chains, causing them to aggregate and thereby increase particle size (Sun et al., 2017). Sun et al. (2020) also confirmed the same results in the study on zeta potential and particle size of Antarctic krill iron chelates.

#### 3.3.2 Thermostability analysis

Fig. 2C and D demonstrated the thermogravimetric analysis curves of TAEDKKLIQ and iron chelate TAEDKKLIQ-Fe. From the TG curves, it was analyzed that the degree of weight loss of the samples varied at different time intervals as the temperature increased (Wang et al., 2025). The progress of weight loss of TAEDKKLIQ and TAEDKKLIQ-Fe was divided into three stages. At the beginning, the primary cause of sample quality loss was the evaporation of moisture. In the second stage, the weight of TAEDKKLIQ was reduced by 60.98 % from 249 °C to 426 °C, and the weight of TAEDKKLIQ-Fe was reduced by 12.32 % by weight from 638 °C to 686 °C. TAEDKKLIQ-Fe exhibited higher initial and final temperatures compared to TAEDKKLIQ, which indicated that its volatility was relatively low. The weight loss of TAEDKKLIQ and TAEDKKLIQ-Fe was about 9.36 % and 4.6 %, respectively, at temperatures up to 800 °C, at which point the remaining material was the amino acid residue from the peptide bond breakage in the peptide (Yang et al., 2024). These results indicate that TAEDKKLIQ-Fe is more thermally stable than TAEDKKLIQ. Possibly due to chelation of ferrous ions, thereby inducing structural changes in the peptide, the breaking of chemical bonds leads to an increase in the energy required (Wang et al., 2025).

#### 3.3.3 UV–visible spectra analysis

**Fig. 3:**
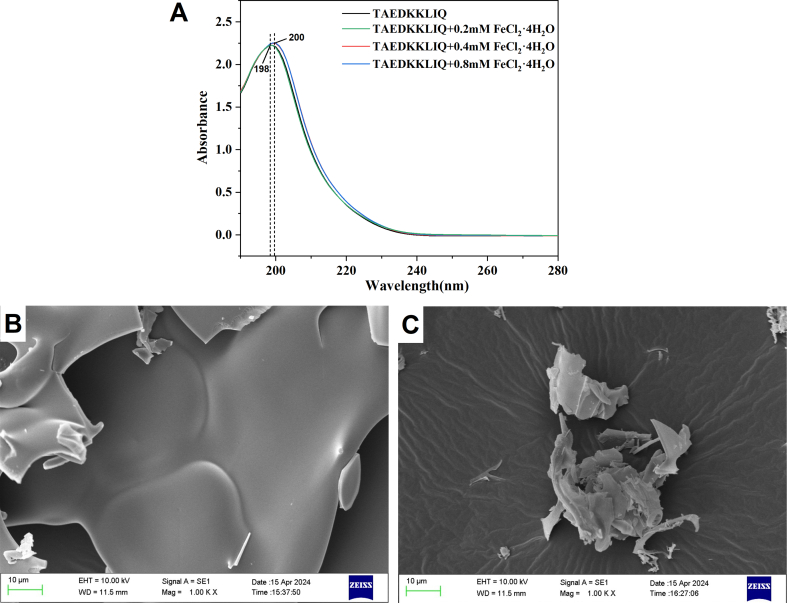
(A) UV–Vis Absorption Spectra of TAEDKKLIQ and TAEDKKLIQ-Fe. (B) Microstructure of TAEDKKLIQ. (C) Microstructure of TAEDKKLIQ-Fe.

The interactions between TAEDKKLIQ and ferrous ions can be shown by changes in the UV–visible spectra (Lin et al., 2021). As shown in Fig. 3A, a strong absorption peak was observed at 198 nm, which was characteristic of the amide bond in peptides. As the Fe^2+^ concentration increased, the peak red-shifted to 200 nm, and the absorbance increased from 2.22 to 2.25, indicating a color enhancement effect. The variation in absorption peak intensity suggested that the chelation between the peptide and ferrous ions induced polarization changes in chromophores (-C=O, -COOH) and co-chromophores (-OH, -NH₂) (Qi et al., 2023). The results showed that TAEDKKLIQ underwent a binding reaction with Fe^2+^ to produce a new substance. Wei et al. (2024) also found similar results in the study of egg yolk peptides with mental ions.

#### 3.3.4 SEM of TAEDKKLIQ and TAEDKKLIQ —Fe

The microstructure of TAEDKKLIQ and TAEDKKLIQ-Fe can be directly observed using SEM. Fig. 3B shows the microscopic surface structure of TAEDKKLIQ observed at a magnification of 1000×. The surface of TAEDKKLIQ was dense and plate-like. After chelation with ferrous ions (Fig. 3C), the dense, flaky structure on the surface of TAEDKKLIQ became loose and smooth. Interactions resulting from metal ion chelation may have disrupted the peptide's inherent compact structure (Zhang et al., 2021). Similar results were observed in scanning electron microscopy of the binding of quinoa protein peptides and iron (Ding et al., 2025). Additionally, the flake structure of TAEDKKLIQ-Fe was larger in size than that of TAEDKKLIQ, which aligns with the particle size results.

### 3.4 Mechanism of TAEDKKLIQ binding to ferrous ions

#### 3.4.1 Fluorescence spectral analysis

**Fig. 4:**
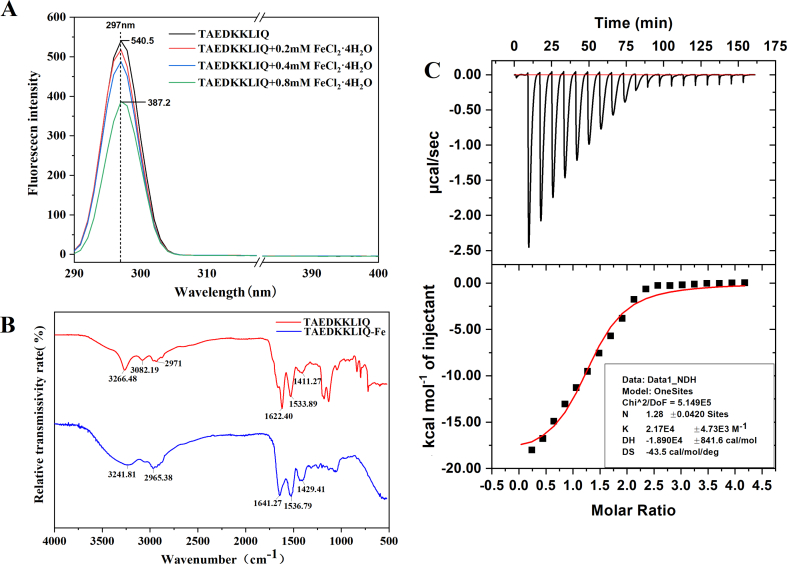
(A) Fluorescence Spectra of TAEDKKLIQ and TAEDKKLIQ-Fe. (B) Infrared spectra of TAEDKKLIQ and TAEDKKLIQ-Fe. (C) Thermodynamic analysis of TAEDKKLIQ binding to Fe^2+^. Data were shown as means ± SEM.

Aromatic amino acids in peptides emit endogenous fluorescence when excited at specific wavelengths, and variations in fluorescence intensity may serve as an indicator of peptide structural changes (Wei et al., 2024). The fluorescence spectroscopy is shown in Fig. 4A. TAEDKKLIQ exhibited the maximum fluorescence intensity at an excitation wavelength of 297 nm. With increasing ferrous ion concentration, the fluorescence intensity at 297 nm decreased, accompanied by a peak redshift. Ding et al. (2025) also observed similar results in their study of the fluorescence spectra of quinoa protein peptides chelated with ferrous ions. It is possible that when TAEDKKLIQ is chelated with ferrous ions, the amino acids or peptide structures in TAEDKKLIQ undergo folding and aggregation, reducing the exposure of aromatic amino acids (Wang et al., 2025).

#### 3.4.2 FTIR analysis

Infrared spectroscopy is commonly used to indicate changes in ligand groups in peptides (Wang, Zhang, Xu, Li and Hao, 2020). The infrared spectra of TAEDKKLIQ and TAEDKKLIQ-Fe were shown in Fig. 4B. In TAEDKKLIQ, the characteristic peaks corresponding to the amide I band (C=O) and amide II bands (N—H and C—N) were 1622.40 cm^−1^ and 1533.89 cm^−1^, respectively. The addition of ferrous ions shifted the peaks to 1641.27 cm^−1^ and 1536.79 cm^−1^, respectively (Yang et al., 2024). The absorption peak of TAEDKKLIQ carboxyl was 1411.27 cm^−1^, while that of TAEDKKLIQ-Fe shifted to 1429.41 cm^−1^, indicating that -COO- may bind with ferrous ions to form -COO-Fe. In the studies by Wei et al. (2024) on the chelation of egg yolk peptides with Fe^2+^ and by Ding et al. (2025) on the chelation of quinoa protein peptides with Fe^2+^, similar changes also appeared. Therefore, the chelation of TAEDKKLIQ with ferrous ions may be realized through the coordination of the N-terminal amino group of the peptide chain or the carboxyl group of the side chain with ferrous ions.

#### 3.4.3 ITC analysis

Isothermal titration calorimetry provides reliable data support for studying the thermodynamic and kinetic properties of peptides chelated with metal ions through automatic titration and thermal detection (Si et al., 2023). The results were shown in Fig. 4C, where the titration of Fe^2+^ with TAEDKKLIQ produced an exothermic binding isotherm, and the ΔH value was negative during the titration process, indicating that the binding process proceeded spontaneously (Sun et al., 2020). Furthermore, the n value of the binding site was 1.19, indicating that the binding of Fe^2+^ molecules to peptides occurs at a single type of binding site. Wang et al. (2020) also drew similar conclusions from the isothermal titration experiments of DHTKE with Zn^2+^, which were consistent with the isothermal titration results of EDLAALEK with Ca^2+^ by Cui et al. (2019). In addition, the binding constant between TAEDKKLIQ and Fe^2+^ was 4.6 × 10^3^. Typically, a binding constant in the range of 10^7^ to 10^8^ indicates a strong reaction. Therefore, the affinity between TAEDKKLIQ and Fe^2+^ was not high, which may help Fe^2+^ to be absorbed and released in the intestine, thereby improving iron bioavailability (Si et al., 2023).

#### 3.4.4 Analysis of intermolecular interaction forces

The process of peptide metal ion chelate formation involves the synergistic effect of multiple intermolecular covalent interactions. Specific chemical denaturants can be used to analyze the functions of various forces during the chelation process: SDS is used to characterize hydrophobic interactions, urea is used to assess the role of hydrogen bonding, NaCl is used to analyze electrostatic interactions, and DTT is used to probe the involvement of disulfide bonds (Liu et al., 2016). These chemical reagents provide an important experimental basis for revealing the molecular mechanism of action of peptide chelation with metal ions.

**Fig. 5:**
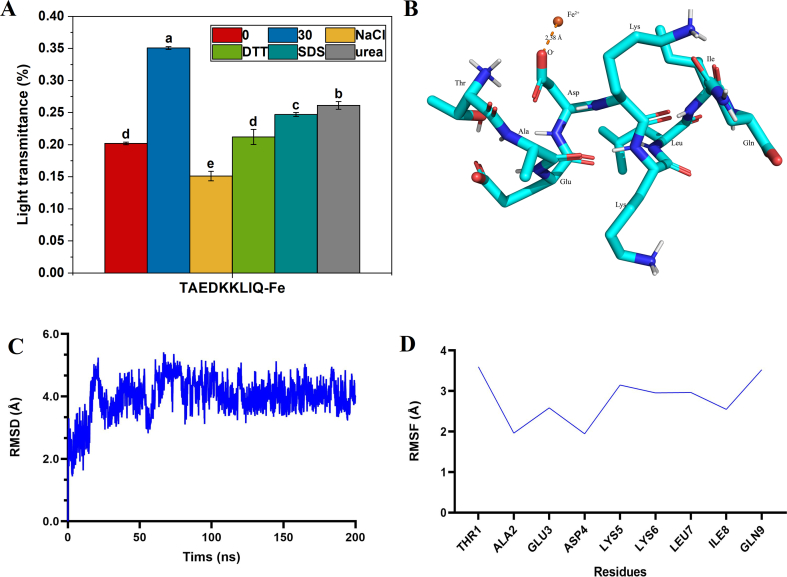
(A)analysis of TAEDKKLIQ-Fe intermolecular interaction forces. (B) Molecular dynamics simulations of TAEDKKLIQ with Fe^2+^. (C) RMSD values of TAEDKKLIQ in the Fe^2+^ environment. (D) RMSF values of TAEDKKLIQ in Fe^2+^ environment.

As shown in Fig. 5A, the light transmission of TAEDKKLIQ-Fe was decreased by the addition of NaCl, SDS, and DTT. In NaCl solution, the transmittance of TAEDKKLIQ-Fe was reduced in all cases, with a decrease in transmittance of 0.20. This suggested that electrostatic interactions were involved in the formation of TAEDKKLIQ-Fe complexes. The results of Bao et al. (2008) on the interaction forces of soybean peptide‑calcium chelates showed that the formation of chelates was electrostatic interactions, which was in agreement with this result. After treatment with urea solution, the light transmittance of TAEDKKLIQ-Fe was all weakened, and the light transmittance was reduced by 0.09, a phenomenon that confirmed that hydrogen bonding interactions were involved in the structural formation of TAEDKKLIQ-Fe chelates. In the presence of SDS solution, the transmittance of TAEDKKLIQ-Fe all showed a decrease in the phenomenon of 0.103, which proved that hydrophobic forces contribute significantly to the structural stabilization of TAEDKKLIQ-Fe chelate. Lv et al. (2013) found that soy peptide bound to calcium through hydrophobic interactions. The transmittance of TAEDKKLIQ-Fe was reduced by 0.138 upon the addition of DTT reagent, which implied that disulfide bonds had a certain effect on chelate formation. By comparing the degree of attenuation of the four forces, it can be concluded that electrostatic interactions played a primary role in the formation of TAEDKKLIQ-Fe and that hydrophobic interactions cannot be neglected (Sun et al., 2020). Meanwhile, hydrogen and disulfide bonds played a secondary role in the formation process of maintaining the stability of TAEDKKLIQ-Fe.

#### 3.4.5 Molecular docking

Molecular docking is extensively employed for forecasting ligand-receptor interactions and pinpointing binding sites (Ding et al., 2025). Thus, molecular docking was adopted to study the potential chelation mechanism of TAEDKKLIQ with Fe^2+^. The results indicated that there were strong binding affinity and compatibility between the two. For the binding mode of TAEDKKKLIQ with Fe^2+^ as shown in Fig. 5B, TAEDKKLIQ was energetically optimized to present a right-handed α-helix structure, and the carboxyl group on the Asp side chain in the TAEDKKLIQ structure formed a stable electrostatic-electrostatic interaction with Fe^2+^ with a bond length of 2.38 Å. This finding was consistent with the results of Hu et al. (2022) on the molecular docking of LVDDHFL with iron molecules, where molecular docking results indicated that the carboxyl group of Asp and the imidazole group of His are considered key factors. Overall, the molecular docking approach provided theoretical support for a potential binding site between Fe^2+^ and TAEDKKLIQ. The results indicated that the chelation of TAEDKKLIQ with Fe^2+^ primarily relied on monodentate coordination involving the carboxyl group of Asp side chain. ITC and FTIR analyses also showed the same results.

#### 3.4.6 Molecular dynamics simulation

RMSD is an important measure of structural stability in molecular dynamics simulations, indicating the displacement of atomic positions from the reference structure during the simulation (Lin et al., 2022). As shown in Fig. 5C, the RMSD value fluctuated greatly in the preliminary stage of molecular dynamics simulation of TAEDKKLIQ-Fe, rapidly increasing from about 1.0 Å to about 3.0 Å. The RMSD value of the receptor-ligand complex was very high at the beginning of the molecular dynamics simulation. This indicated that at the beginning of the simulation, the structure of the receptor-ligand complex changed considerably, and the system was in an unstable state. This may have been due to the gradual structural adjustment of the chelator under the force field to reach an energetically stable state. The mid-term fluctuations during 50–150 ns, with RMSD values fluctuating between 3.0 Å and 5.0 Å with large fluctuations, indicated that the system was in a relatively unstable state during this period of time, probably due to the fact that the interactions between the receptor and ligand were still being adjusted. In the later stage of the simulation from 150 ns onwards, the RMSD value gradually stabilized and the fluctuation amplitude decreased, remaining around 4.0 Å. This indicated that in the later stage of the simulation, the receptor-ligand chelator reached a dynamic equilibrium, and the overall conformational structure gradually stabilized.

As shown in Fig. 5D, the RMSF and interaction with ferrous ions for each amino acid, as well as the initial conformation of TAEDKKLIQ-Fe, the conformation at 50 ns, and the conformation at 200 ns, were predicted. It was found that in the initial molecular docking conformation, the secondary structure of TAEDKKLIQ is in the α-helical state, when only Asp4 and Fe^2+^ formed electrostatic interactions. In the middle stage of the simulation (50 ns), the secondary structure of TAEDKKLIQ was coli, at which time Glu3 and Asp4 formed electrostatic interactions with Fe^2+^; in the late stage of the simulation (200 ns), the system tended to be stabilized, at which time the secondary structure of TAEDKKLIQ remained coli, and the three amino acid residues of Glu3, Asp4 and Gln9 formed electrostatic interactions with Fe^2+^ to form an electrostatic interaction (Lin et al., 2016). The results suggested that the RMSF values of Glu3 and Asp4, which formed electrostatic interactions with Fe^2+^, were lower, while the RMSF value of Gln9 was higher. This may be due to the formation of electrostatic interaction between Gln9 and Fe^2+^ at the early stage of simulation (Zhao et al., 2024). A novel decapeptide isolated and purified from Pacific cod was shown to form a stable chelated structure with Ca^2+^. The molecular dynamics simulation results showed that the oxygen atom of the carboxyl group and the nitrogen atom in the side chain group of this decapeptide were the binding sites (Zhang et al., 2019). These results were similar to those obtained in the present research.

### 3.5 Stability analysis

#### 3.5.1 Temperature and pH stability

**Fig. 6:**
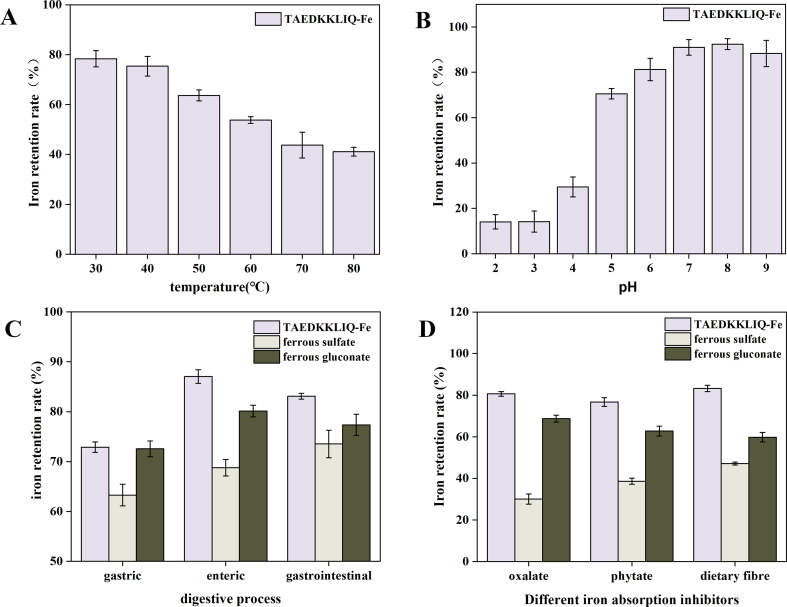
(A) Iron retention rate of TAEDKKLIQ-Fe at different temperatures. (B) Iron retention rate of TAEDKKLIQ-Fe at different pH levels. (C) Iron retention rate in three iron-supplemented preparations under gastrointestinal digestive conditions; (D) Iron retention rate in three iron supplements as affected by different iron absorption inhibitors. Data were shown as means ± SEM.

Fig. 6A showed the effect of temperature variation on ferrous ion retention. The experimental data showed that the ferrous ion content was negatively correlated with temperature in the lower temperature range. The ferrous ions maintained optimal stability at temperatures in the range of 30 °C to 40 °C with no significant loss observed. As the temperature continued to increase, the overall retention of ferrous ions remained above 40 %, indicating that TAEDKKLIQ-Fe was thermally stable, which may be related to the formation of a stable chemical bond between ferrous ions and TAEDKKLIQ (Wei et al., 2024). Qu et al. (2022) conducted an in-depth study on the temperature stability of chelates formed between corn peptides and ferrous ions, and the results showed that the chelates exhibited good thermal stability under various temperature conditions, especially in the temperature range of 10 °C to 60 °C, where the retention of ferrous ions exceeded 85 %.

Fig. 6B shows the pH stability of TAEDKKLIQ-Fe. In the pH range of 6–9, TAEDKKLIQ-Fe was relatively stable with iron retention above 88 %. Especially when the pH value was reduced to 2.0, the iron retention rate of TAEDKKLIQ was only 14.28 %. The reason was that under acidic conditions, excess hydrogen ions competed with ferrous ions for the binding sites of amino acid groups, causing ferrous ions to dissociate (Wu et al., 2019). In contrast, TAEDKKLIQ-Fe was more stable under neutral or alkaline conditions. The pH of the human intestinal environment is approximately 7.2, which means that TAEDKKLIQ-Fe can be stabilized in an alkaline gastrointestinal environment and is in a solubilized state, which allows it to be absorbed more efficiently by intestinal epithelial cells (Zhang et al., 2021). In addition, the pH range of most foods is between 5.0 and 9.0, so theoretically TAEDKKLIQ-Fe could be used as an iron fortifier.

#### 3.5.2 In vitro simulated digestion

Fig. 6C shows the effect of in vitro simulated gastric, intestinal, and gastrointestinal digestion on the iron retention of TAEDKKLIQ-Fe. The results of the gastric digestion simulation experiment showed that the ferrous ion retention of TAEDKKLIQ-Fe was significantly reduced. During simulated gastric digestion, the strongly acidic environment may lead to changes in the chelate structure, while H^+^ in solution competes for binding to the peptide active site, contributing to the dissociation of ferrous ions from the chelate (Hu et al., 2022). Additionally, the spatial conformation of peptides may be disrupted by pepsin hydrolysis, leading to a reduced retention rate of ferrous ions (Zhang, Ding, & Li, 2021). Upon transfer to the simulated intestinal fluid digestion phase, the anions and cations in the system reached a dynamic equilibrium, and chelate stability was significantly enhanced as the pH rose to neutral or weakly alkaline. Under these conditions, previously dissociated ferrous ions were re-coordinated with TAEDKKLIQ to form a stable chelate structure, resulting in a significant increase in ferrous retention (Liu et al., 2023). In addition, the iron retention of TAEDKKLIQ-Fe was superior to that of ferrous sulfate and ferrous gluconate.

#### 3.5.3 The effect of different dietary components on chelates

Daily dietary intake of foods such as cereals and tea is commonly found to contain phytic acid and oxalic acid, as well as being rich in polyphenolic compounds and dietary fiber components. These dietary components have metal chelating properties, which can combine with iron ions to form precipitates, thereby affecting the body's ability to absorb iron and reducing iron bioavailability (Mattar et al., 2022). Therefore, exploring how dietary components affect different types of iron supplements is of non-negligible importance in assessing their bioavailability (Li et al., 2017).

Fig. 6D showed significant differences in soluble iron content among the four iron supplements in the presence of phytic acid, oxalic acid, or dietary fiber alone: TAEDKKLIQ-Fe maintained the highest level, ferrous gluconate was lower, and ferrous sulfate was the lowest. This result confirmed that TAEDKKLIQ-Fe showed superior stability in dietary ingredients compared to conventional iron supplements (ferrous gluconate and ferrous sulfate). The addition of dietary ingredients reduced the absorption of metal ions (Hu et al., 2022), and other studies showed that iron peptide complexes protect iron ions from dietary ingredients (Ramesh et al., 2022). Fig. 6D further showed that among the three dietary components, phytic acid had the most significant inhibitory effect on TAEDKKLIQ-Fe, which maintained the retention of soluble iron in iron chelates at 76.73 %. Phosphate groups in phytic acid appear to bind more strongly to iron ions (Lin et al., 2022). Overall, TAEDKKLIQ-Fe was more effective in counteracting the inhibition of iron absorption by food components such as phytic acid, thus enhancing the bioavailability of iron in the body.

### 3.6 Cell viability assay analysis

MTT assay was used to assess the effects of TAEDKKLIQ-Fe and lactoferrin on the viability of Caco-2 cells, and the results were shown in Table 2. Within the concentration range of 0.125–1 mg/mL, the viability of Caco-2 cells treated with lactoferrin showed a significant decreasing trend prior to that of TAEDKKLIQ-Fe. When the concentration of TAEDKKLIQ-Fe reached 2 mg/mL, cell viability significantly decreased (*P <* *0.05*). However, even when the concentration of TAEDKKLIQ-Fe was increased to 4 mg/mL, cell viability remained above 80 % (Moreda-Pineiro et al., 2020). A study reported that the survival rate of Caco-2 cells treated with whey peptide iron chelates exceeded 87 % (Caetano-Silva et al., 2018), consistent with the present results. In contrast, when lactoferrin concentration increased to 4 mg/mL, cell viability dropped below 60 %. This suggested that the peptide iron chelate had no significant toxic effects on cell growth and produced fewer side effects on cells than lactoferrin. This provided important evidence for the subsequent safety assessment of chelates.

**Table 2:** Effect of TAEDKKLIQ-Fe on the viability of Caco-2 cells.

Group	Concentration (mg/mL)	Relative vitality of cells (%)	
		TAEDKKLIQ-Fe	lactoferrin
1	0.125	98.06 ± 1.22	92.86 ± 1.03
2	0.25	97.72 ± 1.67	81.43 ± 0.94
3	0.5	97.53 ± 0.89	77.76 ± 1.27
4	1	97.21 ± 1.45	74.83 ± 0.84
5	2	92.24 ± 1.43	66.90 ± 1.42
6	4	83.78 ± 1.32	53.73 ± 0.94

Note: Data were shown as means ± SEM.

## 4 Conclusion

In this study, a novel ferrous ion chelating peptide (TAEDKKLIQ) was identified from chicken blood hemoglobin by isolation and purification. The structure of the chelate complex TAEDKKLIQ-Fe was characterized. And molecular docking and molecular dynamics simulations were combined to further determine the binding mechanism between TAEDKKLIQ and ferrous ions. The results indicated that the structure of the peptide changed after chelation with ferrous ions, and the particle size increased significantly. Additionally, the study revealed that TAEDKKLIQ possessed a single ferrous ion binding site, where the Asp residue served as the primary coordinating group, forming a monodentate coordination with Fe^2+^. And hydrogen bonds, and disulfide bonds also play important roles. In addition, the TAEDKKLIQ-Fe chelate exhibited excellent thermal and pH stability, maintaining structural integrity and high iron-retention capacity under neutral and mildly alkaline conditions. The structural stability of the chelate was maintained during in vitro simulated gastrointestinal digestion, and its superior digestive resistance and potential bioavailability were demonstrated. These studies indicated that the chelate exhibited distinct advantages in terms of chemical structural stability, digestive tolerance, and safety, indicating its potential viability as an iron fortifier. This provided a critical chemical basis and theoretical support for the application of animal-derived peptide–iron chelates in food fortification and functional food development. Further research can focus on its absorption and metabolic efficiency within the body, as well as product development, to assess its practical application potential in the food industry.

## CRediT authorship contribution statement

**Hanyu Guo:** Writing – original draft, Formal analysis. **Ying Zhou:** Data curation. **Cancan Luo:** Investigation. **Zhiyu Li:** Software. **Jiulan Peng:** Investigation. **Weimin Xu:** Project administration. **Daoying Wang:** Supervision. **Jing Yang:** Writing – review & editing, Project administration, Conceptualization.

## Declaration of competing interest

The authors declare that they have no known competing financial interests or personal relationships that could have appeared to influence the work reported in this paper.

## Data Availability

Data will be made available on request.
