# Corrigendum to “The binding mechanism of a novel ferrous ion chelating peptide from chicken blood hemoglobin and the bioavailability of the chelate” [Food Chem. X 32 (2025) 103349]

**DOI:** 10.1016/j.fochx.2026.103578

**Published:** 2026-01-30

**Authors:** Hanyu Guo, Ying Zhou, Cancan Luo, Zhiyu Li, Jiulan Peng, Weimin Xu, Daoying Wang, Jing Yang

**Affiliations:** a Institute of Agro-product Processing, Jiangsu Academy of Agricultural Sciences, Nanjing, Jiangsu 210014, PR China; b School of Life Sciences, Anhui Normal University, Wuhu, Anhui 241001, PR China; c School of Life Sciences and Food Engineering, Huaiyin Institute of Technology, Huaian, Jiangsu 223003, PR China

The authors regret “Section 3.3.9, paragraph 2, “As shown in Fig. 5D and E"; Figure captions, Fig. 5, “(E). The modes (conformations) of TAEDKKLIQ-Fe complex at 0 ns, 50 ns, and 200 ns.”

Fig. 5

**Figure:**
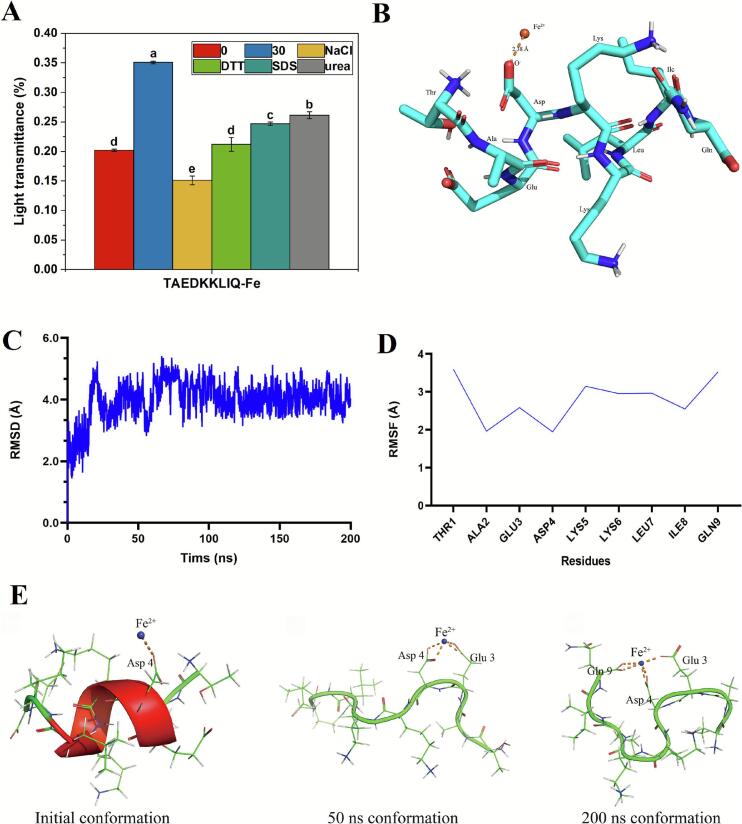

The authors would like to apologise for any inconvenience caused.

